# Surveillance of vector populations and malaria transmission during the 2009/10 El Niño event in the western Kenya highlands: opportunities for early detection of malaria hyper-transmission

**DOI:** 10.1186/1756-3305-4-144

**Published:** 2011-07-22

**Authors:** Ednah N Ototo, Andrew K Githeko, Christine L Wanjala, Thomas W Scott

**Affiliations:** 1 Kenya Medical Research Institute, Centre for Global Health Research, Climate and Human Health Research Unit. P.O. Box 1578, Kisumu, Kenya; 2 Egerton University, P.O. Box 536, Njoro, Kenya; 3 University of California, Davis, California 95616, USA

## Abstract

**Background:**

Vector control in the highlands of western Kenya has resulted in a significant reduction of malaria transmission and a change in the vectorial system. Climate variability as a result of events such as El Niño increases the highlands suitability for malaria transmission. Surveillance and monitoring is an important component of early transmission risk identification and management. However, below certain disease transmission thresholds, traditional tools for surveillance such as entomological inoculation rates may become insensitive. A rapid diagnostic kit comprising *Plasmodium falciparum *circumsporozoite surface protein and merozoite surface protein antibodies in humans was tested for early detection of transmission surges in the western Kenya highlands during an El Niño event (October 2009-February 2010).

**Methods:**

Indoor resting female adult malaria vectors were collected in western Kenya highlands in four selected villages categorized into two valley systems, the U-shaped (Iguhu and Emutete) and the V-shaped valleys (Marani and Fort Ternan) for eight months. Members of the *Anopheles gambiae *complex were identified by PCR. Blood samples were collected from children 6-15 years old and exposure to malaria was tested using a circum-sporozoite protein and merozoite surface protein immunchromatographic rapid diagnostic test kit. Sporozoite ELISA was conducted to detect circum-sporozoite protein, later used for estimation of entomological inoculation rates.

**Results:**

Among the four villages studied, an upsurge in antibody levels was first observed in October 2009. *Plasmodium falciparum *sporozoites were then first observed in December 2009 at Iguhu village and February 2010 at Emutete. Despite the upsurge in Marani and Fort Ternan no sporozoites were detected throughout the eight month study period. The antibody-based assay had much earlier transmission detection ability than the sporozoite-based assay. The proportion of *An. arabiensis *among *An. gambiae s.l*. ranged from 2.9-66.7% indicating a rearrangement of the sibling species of the *An. gambiae s.l *complex. This is possibly an adaptation to insecticide interventions and climate change.

**Conclusion:**

The changing malaria transmission rates in the western Kenya highlands will lead to more unstable transmission, decreased immunity and a high vulnerability to epidemics unless surveillance tools are improved and effective vector control is sustained.

## Background

Malaria epidemics occurred in the western Kenya highlands in the 1930-40s and then disappeared until the late 1980s [1,2]. Both periods are associated with anomalous warming and precipitation. Epidemics caused severe morbidity and mortality in the 1990's onwards, and as a consequence interventions to control transmission and disease were initiated between 2003-2006 through the use of insecticide impregnated bed nets, indoor residual spraying (IRS) and artemisinin combination therapies (ACTS) [3]. High malaria transmission rates were reported prior to 2005 and this varied in the different ecological setting in the highlands [4,5]. In 2006, large scale distribution of free long lasting treated bed nets (LLNs) was undertaken resulting in substantial reduction in transmission [6]. For example, malaria transmission control in one site in the western Kenya highlands reduced indoor densities of *Anopheles gambiae *by 98% and *Anopheles funestus *by 85% [7]. While vector control is having a high impact on transmission, the human population is less exposed to the disease and this could lead to decline in immunity and subsequent vulnerability to malaria epidemics. It is critical that as vector control scales up, monitoring of trends in transmission is undertaken continuously to detect and contain any transmission upsurges. However, under very low transmissions, the current methods of detecting sporozoite infections in vectors become unreliable as few vectors may be detected. It has been shown that in some sites in the highlands of western Kenya, elimination of malaria is in sight following intense vector control using IRS and LLNs [8].

Vector control using ITNs and LLNs has been shown to selectively suppress populations of the more anthrophilic and endophilic *An. gambiae s.s*. leading the more zoophilic *An. arabiensis *to predominate and maintain transmission [9,10].

We undertook a short study to examine the possibility of using a rapid diagnostic kit for the detection of anti-malaria immune markers circum-sporozoite protein antibodies (CSP) and merozoite surface protein antibodies (MSP) as an early indicator of transmission changes in human populations living in contrasting eco-epidemiological and transmission setting in the western Kenya highlands during the 2009 El Niño event. These ecosystems are comprised of both poorly drained and well-drained valleys. In addition, a profile of the *An. gambiae *s.l. sibling species was undertaken to provide the baseline for future comparative studies.

## Materials and methods

### Study site description

**Figure 1:**
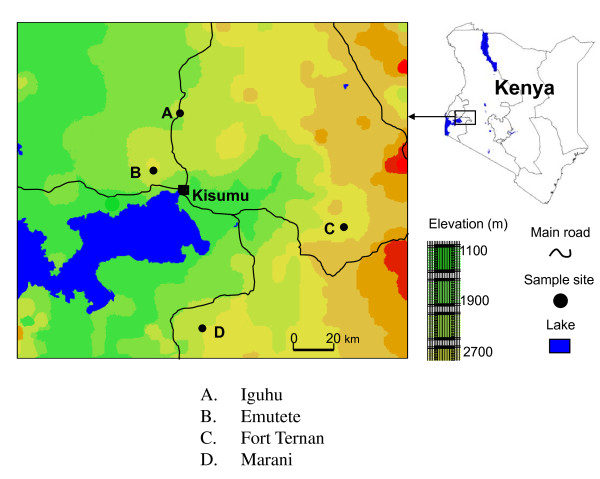
**Map of the study sites in the Western Kenya Highlands**.

The study was carried out in four sites defined by the drainage type and level of malaria transmission. Emutete in Vihiga district (0°.026'N; 34°.64'E and elevation 1,506 m above sea level and Iguhu in Kakamega (0°.17'N; 34°.74'E, and elevation 1,450-1,580 m above sea level are sites with poor drainage and high malaria transmission. Conversely, Fort Ternan in Kericho (0°.12'S; 35°.21'E and elevation 1,500-1,600 m above sea level and Marani in Kisii (0°.02'N; 34°.48'E, elevation 1,520-1,700 m above sea level have good drainage, low and unstable malaria transmission (Figure 1). The western highlands experience two rainy seasons one being long (March-May) and the other being short (October-November).

Iguhu and Emutete sites have large flat-bottomed valleys with slow moving rivers. These sites are referred to as U-shaped valleys; ecosystems which support several vector breeding habitats. Fort Ternan and Marani sites have narrow shaped valleys, small flat surface areas and fast flowing rivers. These sites are referred to as V-shaped valleys; ecosystems that have few suitable vector breeding habitats.

### Sample size calculation

Because the malaria prevalence and CSP-MSP antibody prevalence in the study area was not well known, the sample size calculation was carried out assuming the prevalence was 50% for both parameters. The sample size was calculated to achieve a 95% confidence and precision level of 5%.

The sample size required was 384 children from all the study sites, i.e. 77 children per site. Assuming the truancy of 10% a total sample size of 425 children was obtained from 600 children (120 from each site) who had given consent to participate in the study. The homes of the 600 children were geo-referenced, mapped and tested for clustering [11]. No clustering was found, and the homes were therefore randomly distributed. The human population density in all the study sites is very similar and so equal numbers of children were allocated to each site. Of the final sample size of 425 children each site was allocated 85 children.

### Study Population

Cohorts of 170 children in the two U-shaped valleys, 170 children in the two V-shaped valleys aged 6-15 years were recruited for monthly *P. falciparum *CSP-MSP antibody prevalence surveys for 16 months. Eight months data was extracted (from September 2009- April 2010) to coincide with the entomological data collection. Before the children were allowed to participate in the study, consent was obtained from their parents or guardians. Children aged between 6-15 years residing in the study sites and with no reported chronic illness except malaria were allowed to participate in the study. Children who were found having fever at the time of sampling were taken to the nearest government clinic for treatment.

### Testing for CSP and MSP antibodies

After swabbing the finger with an alcohol pad, blood from a finger prick was collected in microvet tubes, transported to the Kenya Medical Research Institute laboratories and span for 5 minutes at 1800 rounds per minute for serum separation. The rapid malaria diagnostic kit from SCIMEDX ((SCIMEDX Corporation, Deville, New Jersey, USA) contains two recombinant capture *Plasmodium falciparum *and *Plasmodium vivax *parasite antigens circumsporozoite protein (CSP) and merozoite surface protein (MSP). The immunochromatographic rapid test qualitatively detects antibodies of all isotypes (IgG, IgM and IgA) specific to *Plasmodium falciparum *and *Plasmodium vivax*. The recombinant malaria *P. falciparum *antigen (CSP-MSP) and *P. vivax *antigen (CSP-MSP) colloid gold conjugate and serum sample moves along the test membrane chromatographically to the test region and forms a visible line of the antigen-antibody-antigen gold particle complex. The test has high sensitivity (91.3%) and specificity (98.5%). Results were scored after 10 minutes as positive or negative. Reactions occurring after 20 minutes were rejected. The tests were only carried out for *P. falciparum *as *P. vivax *is not known to occur in the study area.

### Mosquito collection and identification

Adult mosquitoes were collected monthly from September 2009 to April 2010 by the pyrethrum spray sheet method [12] in 10 houses per site. The number of sleepers in each house was recorded. The collections were transported to the laboratory and female vector morphologically identified as *Anopheles gambiae s.l *or *Anopheles funestus *[13]. The females were then classified according to their gonotrophic stages. Legs and wings of females of *Anopheles gambiae *s.l were frozen at -20°C in labelled vials before molecular identification by PCR into *Anopheles gambiae *or *Anopheles arabiensis *according to Scot *et al*. [14].

### Sporozoite infection detection (ELISA)

The head and thorax of adult females were separated from the abdomen, placed in 5 ml Epperndoff tubes, macerated and the triturate used for sporozoite ELISA according to Wirtz *et al*. [15].

### Calculation of the entomological inoculation rates (EIR)

The entomological inoculate rate (*mas*) is the product of the man biting rate (ma) multiplied by the proportion of sporozoite infected female vectors (s) [16]. The fraction of human biting females was obtained from the blood-fed fraction of the PSC collections [17]. The man biting rate was obtained by dividing the number of blood fed females per house by the number of sleepers. The half-gravid fraction was not included in the calculation as they could not have fed on house occupants in the night before collection. Blood digestion takes longer in the cool highlands. It was assumed that > 90% of the *An. gambiae s.l*. females had fed on humans [18,19]. None of the 28 *An. arabiensis *were sporozoite positive by ELISA therefore this species was not included in the EIR calculation.

### Rainfall data

Monthly rainfall, maximum and minimum date for the period Jan 2009-April 2010 was obtained from the Kenya Department of meteorology.

### Data management and analysis

Data was entered into Microsoft Excel spread sheets. Statistical analysis was carried out using Excels Analysis ToolPak. The difference in the mean indoor vector densities was tested by a two tail t-test and a regression analysis was used to determine the strength of the relationship between the mean indoor resting densities and mean prevalence of CSP and MSP antibodies.

### Ethical approval

Scientific and ethical clearance was given by Kenya Medical Research Institute. An inclusion criterion was the provision of informed consent.

## Results

An El Niño event had been predicted to occur in East Africa in 2009/10. El Niño is characterized by heavy rains in the months of November and December. Anomalous temperatures may prevail during this period. Such conditions may lead to rapid vector breeding, high malaria transmission and epidemics in the western Kenya highlands.

**Figure 2:**
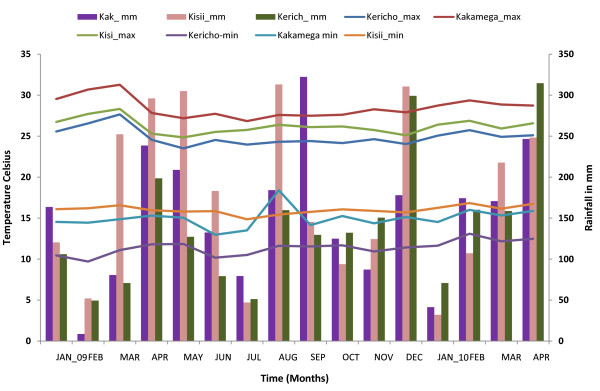
**Rainfall and Temperature patterns of the study sites in western Kenya Highlands**.

Data obtained from the Kenya Department of meteorology indicated heavy rains in all study sites in December 2009. The Kakamega station reported 178 mm, Kericho, 299 mm and Kisii, 310 mm (Figure 2). These levels of rainfall were expected to trigger an increase in vector densities. Kakamega reported high rainfall (322 mm) in September 2009 and 87 mm in November. Rainfall declined to less than 100 mm mean monthly rainfall in all the stations in January 2010. Anomalous temperatures were not observed (Figure 2).

### Vector populations

*Anopheles gambiae *and *Anopheles funestus *were the only vectors collected with the former comprising 98.7% (226/229) of the population. *Anopheles funestus *was not collected in the V-shaped ecosystems of Fort Ternan and Marani in Kisii district. Only the data for *An. gambiae s.l*. will be reported.

**Figure 3:**
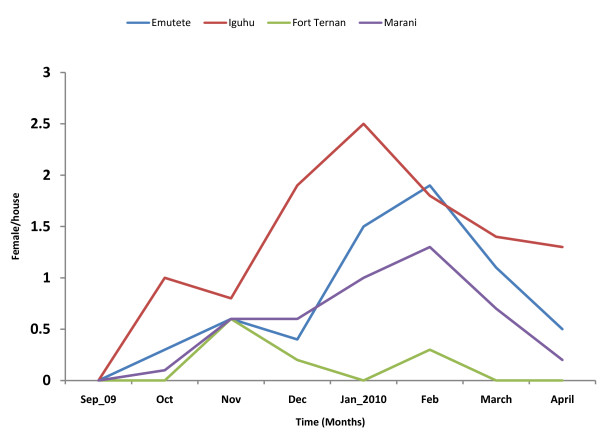
**Indoor resting densities of *An. gambiae *across the study sites**.

The U-shaped ecosystems had 3-fold more vectors compared to the V-shaped ecosystem, this being consistent with the availability of breeding habitats. Heavy rainfall in Kakamega triggered a surge in the *An. gambiae s.l*. population from a mean monthly indoor resting density of zero in September 2009 to 2.5 females per house in January 2010 (Figure 3). A similar trend was observed in Emutete with the population peaking in February 2010. The *An. gambiae s.l*. population in the V-shaped Fort Ternan had low response to rainfall with the highest indoor resting density (0.6 females/house) being observed in November 2009. In Marani a surge of 0.6 females per house was observed in November followed by a peak of 1.3 females/house in February 2010 (Figure 3). Despite continued rainfall in February to April 2010 the vector populations continued to decline. Within the U-shaped ecosystems the indoor resting densities were not statistically different (t-two tail = 0.073); however, the indoor resting densities in V-shaped ecosystem were significantly different (t-two tail = 0.025).

### Entomological inoculation rates

Among the U-shaped ecosystems the daily entomological inoculation rate at Emutete was 0.006 infected bites/person (ib/p) and in Iguhu 0.01 ib/p. In the V-shaped ecosystem (Fort Ternan and Marani) no transmission was detected during the eight months period (Table 1). At Iguhu, transmission was detected in December 2009 and January 2010, while at Emutete transmission was detected only in February and March 2010. The estimated annual EIR at Iguhu was 3.68 ib/p/yr and at Emutete 2.05 ib/p/yr. The mean sporozoite rate observed at Iguhu was 3.1 and at Emutete 2.8 (Table 1).

**Table 1:** Annual EIR of the study sites in Western Kenya Highlands

	U-shaped valley		V-shaped valley	
	**Emutete**	**Iguhu**	**Fort Ternan**	**Marani**

**Mean sporozoite rate**	2.8	3.1	0	0

**Monthly EIR**				
Sept_09	0.00	0.00	0.00	0.00
Oct	0.00	0.00	0.00	0.00
Nov	0.00	0.00	0.00	0.00
Dec_09	0.00	0.02	0.00	0.00
Jan_10	0.00	0.06	0.00	0.00
Feb	0.03	0.00	0.00	0.00
March	0.01	0.00	0.00	0.00
April	0.00	0.00	0.00	0.00

Daily EIR	0.006	0.01	0.000	0.000

Annual EIR	2.05	3.7	0.00	0.00

### CSP-MSP antibodies and *P. falciparum *dynamics

**Figure 4:**
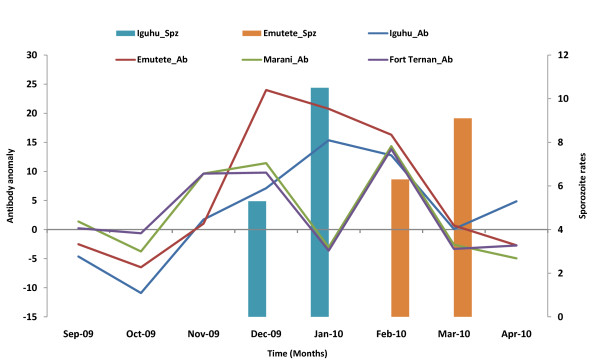
**Comparisons between CSP-MSP antibodies and EIR in all the study sites**.

An upward trend in the prevalence of CSP-MSP antibodies was observed in October 2009. Figure 3 shows the departure of the monthly prevalence from the long term mean calculate from 16 months of data. The increase in antibody prevalence was 12.6% in Iguhu, 7.5% in Emutete, 13.4% in Marani and 10.3% in Fort Ternan. At Emutete the peak prevalence (24%) was observed in December 2009 and at Iguhu (15.3%) in January 2010. After a fairly linear and continuous increase in antibody prevalence a downward trend in prevalence was observed until March 2010. In the V-shaped eco-systems of Fort Ternan and Marani the change in prevalence was characterized by a peak in December 2009 (approx 10%), a dip in January 2010 (-3%) and then a rise again in February 2010 (approx 14%). A decrease in antibody prevalence continued until March to the same levels as the U-shaped ecosystem (Figure 4).

In Iguhu *P. falciparum *sporozoites were detected in December 2009 and January 2010 and in Emutete February and March 2010. No sporozoites were detected in the V-shaped ecosystem (Fort Ternan and Marani). Increase in transmission indicated by the CSP-MSP antibody prevalence was detected in November 2009 in all sites. First detection of sporozoite transmission at Iguhu occurred in December 2009 while in Emutete it was not detected until February 2010. Thus the CSP-MSP is a more sensitive indicator of transmission than that of the entomological method (Figure 4). Significant correlation was observed between indoor resting densities of *An. gambiae s.l*. and CSP_MSP prevalence in the four sites (Table 2).

**Table 2:** The relationship between monthly indoor resting densities *of An.gambiae s.l *and the prevalence CSP_MSP antibodies in the human population

Site	Adjusted R-Squired	p value
Iguhu*	0.567	0.031
Emutete	0.071	0.282
Fort Ternan*	0.570	0.030
Marani	0.159	0.204

*Significant correlation between indoor resting densities of *An. gambiae *and CSP_MSP prevalence.

### Distribution of *An. gambiae *and *An. arabiensis*

The highest proportion of *An. arabiensis *was observed in Fort Ternan (66.7%) followed by Iguhu (6.7%) and Marani (3.6%). The lowest proportion was observed in Emutete (2.9%). Species distribution was not related to the type of the ecosystem (Table 3). Fort Ternan is close to the Kericho meteorological station which has a mean annual rainfall of 2019 mm while Marani has a mean of 2100 mm as indicated by the Kisii meteorological station. Differences in rainfall cannot explain the differences in the proportion of *An. arabiensis *in the two sites. Moreover, the Fort Ternan and Marani sites have similar coverage of insecticide treated bed nets (70%, Githeko unpublished data). Ten years ago, no *An. arabiensis *were detected in western Kenya at altitude above 1400 m above sea level [20].

**Table 3:** *An. gambiae s.s*. and *An. arabiensis *distribution in four sites in Western Kenya (PCR)

	U-shaped valley		V-shaped valley		
		
Species	Emutete	Iguhu	Fort Ternan	Marani	Mean proportion
Total identified	35	89	9	28	

*An. gambiae*	97.1	93.3	33.3	96.4	91.3

*An. arabiensis*	2.9	6.7	66.7	3.6	8.7

## Discussion

Malaria in the highlands of western Kenya is characterized by unstable transmission that is closely related the ecosystem type and weather variability. The two ecosystems (U and V-shaped) have different vulnerabilities to malaria epidemics. Current interventions using long lasting insecticide treated nets (LLINs) and in some sites indoor residual spraying (IRS) are suppressing vector populations and also selecting the less anthropophilic member of the *An. gambiae sl*. complex, *An. arabiensis *[9]. Decreasing transmission due to reduced vector abundance and dominance of less efficient species will result in reduced exposure to malaria parasites and fewer individuals with immunity to malaria [21] with subsequent increased vulnerability to epidemics and severe malaria. These changes in transmission will require careful monitoring and surveillance as part of early detection of transmission risks and the evolution of epidemics.

Heavy rains associated with the 2009/2010 El Nino event increased vector population in the entire study area resulting in peaks in January and February 2010. *Plasmodium falciparum *sporozoites were detected in December and January at Iguhu and February and March at Emutete and none in the V-shaped ecosystems (Marani and Fort Ternan). However, in October 2009 the prevalence of antibodies increased in all the sites suggesting exposure of the population to the parasite. In Emutete the peak of antibody prevalence was observed in December 2009 while sporozoites were first detected in February 2010. Analysis of the relationship between the indoor resting densities of female *An. gambiae *and the prevalence of antibodies revealed a significant correlation between the two variables at Iguhu and Fort Ternan but not at Emutete and Marani. This may have resulted from under-sampling of the vectors in the former sites. The human population sampled had a wider dispersion compared to the houses sampled for vectors.

Assessment of the abundance showed that the U-shaped ecosystem had 3-fold higher vector densities in the V-shaped valleys and this is supported by the higher prevalence of antibodies in the U-shaped ecosystem.

These results indicate that surveillance of CSP-MSP antibodies provided an earlier and more responsive indicator to malaria transmission than EIR. The rapid diagnostic kit (RDT) is cheap and easy to use. A large human population can be assessed in a very short time and results are available immediately. The RDT provides a new opportunity for early detection of hyper-transmission and may complement results of the climate based early malaria epidemic prediction models [22].

In 1998, *An. gambiae *was the only member of the *An. gambiae *complex reported in the western Kenya highlands at 1500 m asl [23]. In another study *An. arabiensis *was not detected in areas above 1400 m asl in the western Kenya highlands [20]. From June 2003-June 2004; no *An. arabiensis *were detected at Marani while the proportion of this species at Iguhu was 0.8% [5]. Our results indicate that the proportion of *An. arabiensis *at Iguhu has increased to 6.7% and at Marani to 3.6%. More remarkable was the high proportion of this species that was observed at Fort Ternan (66.7%). The ownership of LLINs in Marani and Fort Ternan are similar and therefore difference in the LLIN ownership cannot explain the differences in the proportion of *An. arabiensis *observed in the two sites. A contemporary Larval ecology study at Fort Ternan reported a 71% proportion of An. arabiensis in the An. gambiae s.l. population at Fort Ternan [24]. In the lowlands of western Kenya *An, gambiae *has been largely replaced by *An. arabiensis *and this has been attributed to the wide scale use of LLINs [9,10]. The continued use of LLINs in the western Kenya highlands and continued warming due to climate change will favor the more zoophilic and possibly exophilic *An. arabiensis*. While this species is less amenable to vector control using insecticides it is a much less efficient vector. It is capable of maintaining low level transmission but has been known to be the only vector causing epidemics particularly in the semi arid ecosystems. To date *An. arabiensis *is the only reported vector in the central Kenya highlands [25].

While the CSP-MSP antibody profiles in the human population were very similar in Marani and Fort Ternan, the vector profile was not similar with For Ternan. This is indicative of lower indoor resting densities. One possibility is that a large population of *An. arabiensis *females in Fort Ternan was not resting indoors.

## Conclusion

This study indicated that antibodies to malaria parasites in the human population CSP-MSP are more sensitive to changes in transmission than the traditional EIR and that they have the potential to detect epidemic threats better. The changing vectorial system may favour *An. arabiensis *which may pose challenges in vector surveillance due to its zoophilic and exophilic behaviour. The changing malaria transmission rates in the western Kenya highlands will lead to more unstable transmission, decreased immunity and a high vulnerability to epidemics unless surveillance tools are improved and effective vector control is sustained. More detailed research is required in this area to improve sampling precision and long-term data sets so that the utility of the antibody surveillance tool can be confirmed.

## Competing interests

The authors declare that they have no competing interests.

## Authors' contributions

ENO participated in the design of the study, conducted data collection, statistical analysis and drafting of the manuscript. AKG participated in the design and coordination of the study, facilitated field sample collection, interpretation of the data and drafting of the manuscript. CLW participated in data collection and analysis of data. TS participated in the study coordination. All authors approved the manuscript for submission.
